# 2611. Risk Factors Associated with Longer Hospital Stay in Adult Patients with Respiratory Syncytial Virus Infections

**DOI:** 10.1093/ofid/ofad500.2225

**Published:** 2023-11-27

**Authors:** Ashish Bhargava, Ambreen Malik, Susan M Szpunar, Leonard B Johnson, Mamta Sharma

**Affiliations:** Ascension St. John Hospital; Wayne State University School of Medicine, Grosse Pointe Woods, Michigan; Ascension St John Hospital, Warren, Michigan; Ascension St. John Hospital, MI; Ascension St. John Hospital, MI; Ascension St John Hospital & Medical Center, Grosse Pointe Woods, MI. 48236, Michigan

## Abstract

**Background:**

Respiratory Syncytial Virus (RSV) is known to cause severe disease in elderly individuals and patients with underlying cardiopulmonary or immunocompromised conditions. Little is known about the factors that are associated with the length of hospital stay (LOS).

**Methods:**

A multicenter historical cohort study was conducted on adult patients hospitalized for laboratory-confirmed RSV-related diseases in Ascension hospitals in Southeast Michigan between January 2017 and December 2021. Hospitalized patients were identified using ICD 10 codes for RSV-related diseases. Electronic medical records were reviewed after IRB approval. LOS was categorized as below the mean LOS or at the mean or above. Data were analyzed using Student’s t-test, the chi-squared test, the Mann-Whitney U test and logistic regression using SPSS v. 29.0.

**Results:**

Of 360 patients, the mean (sd) age of the cohort was 69.9 + 14.7 years, 228 (63.5%) were female and 227 (63%) were white. The mean body mass index (BMI) was 30.6 + 9.7 kg/m^2^ . The mean Charlson Weighted Index of Comorbidity (CWIC) score was 2.2 + 1.9. The mean hospital LOS was 7.1 + 5.4 days. Factors associated with longer hospital LOS (< =7) in univariable analysis were older age on admission, smoking status, CWIC score, home oxygen, encephalopathy, oxygen requirement during hospitalization, qSOFA score, lower respiratory tract infection, abnormal chest imaging, acute kidney injury, liver injury and respiratory failure requiring intubation. The multivariable logistic regression revealed that predictors for prolonged hospital LOS were age on admission (odds ratios [OR], 1.034; 95% CI 1.01-1.06), smoking status (OR, 2.2; 95% CI 1.2-3.9), CWIC (OR, 1.1; 95% CI 0.99-1.30), liver injury (OR, 4.7; 95% CI 1.16-19.33), oxygen requirement during hospitalization (OR, 2.9; 95% CI 3.02-7.72). and respiratory failure requiring intubation (OR, 9.9; 95% CI 3.02-39.20).

**Conclusion:**

Our study finds that age on admission, patient’s smoking status, their CWIC, liver injury, oxygen requirement during hospitalization and respiratory failure requiring intubation were significantly associated with prolonged hospital LOS among adult patients with RSV infection. Every one unit increase in age and CWIC increased the risk for prolonged hospital LOS by 3.4% and 13.9% respectively.

**Disclosures:**

**All Authors**: No reported disclosures

